# Entropy Optimization by Redesigning Organization in Hospital Operations

**DOI:** 10.3390/e25101447

**Published:** 2023-10-14

**Authors:** Windi Winasti, Hubert Berden, Frits van Merode

**Affiliations:** 1 IQ Healthcare, Radboudumc, 6525 EP Nijmegen, The Netherlands; b.berden@etz.nl; 2 Elisabeth-TweeSteden Ziekenhuis, 5022 GC Tilburg, The Netherlands; 3 Care and Public Health Research Institute, Maastricht University, 6200 MD Maastricht, The Netherlands; f.vanmerode@maastrichtuniversity.nl; 4 Maastricht University Medical Centre+, 6229 HX Maastricht, The Netherlands

**Keywords:** hospitals, organization, information processing, coordination, nurses, flexibility, entropy

## Abstract

A redesign of hospitals (i.e., partitioning departments and delegating decision authority) may be needed to deal with variable demand. Uncertain demands and throughput times often need short reaction times. In this study, we develop quantitative methods to guide a redesign through an information-processing approach. To demonstrate how the methods can be used in practice, we tested them by applying them to a large perinatology care system in the Netherlands. We used the following two methods: 1. portfolio optimization and 2. efficient coordination of workload and reallocation of nurses. Our case study of a large perinatology care system showed that several designs of clustered units minimized the demand uncertainty in the perinatology care system. For the coordination strategy, the information and decision uncertainty is minimized when the decision power is positioned at the operation level and with the help of a centralized information system. When the operation decision-making power is not supplemented with the centralized and system-wide information system, hospitals can better use the hierarchy model, where the manager holds decision-making power with a system-wide overview. We also found that the speed of decision-making in real-time depends on the level of information aggregation set up by the system. We conclude that combining the correlation perspectives and the entropy theory is a way of quantifying how organizations can be (re)designed.

## 1. Introduction

Uncertainty and variability make it difficult to plan the right number of resources, such as nurses [1,2,3]. When understaffing occurs, new patients may be turned away, and overstaffing is often considered to be inefficient. The development of nurse capacity management for a short-term period is an essential step in developing the real-time control of hospital resources, given the constraints of the organization as it is [4]. The organization of real-time decision-making is becoming an interesting topic for hospital practitioners [5,6].

Capacity planning and control processes in hospitals are structured as being strategic, tactical, operational, and real time (also referred to as operational online) [7,8]. Decisions made earlier will govern the planning options and constraints at subsequent levels. Mismatches between resources and patient demand in real-time can be alleviated if proper decisions regarding how to deal with variability are decided strategically. As was discussed by Van Merode et al. [9], the allocation of resources during strategic planning should consider fluctuations in demand. Hospitals may add a certain amount of slack capacity (i.e., buffer) to the designated capacity per department, depending on the risk the decision-maker is willing to accept when the patient demand cannot be fulfilled [9]. The strategic planning then decides the amount of required slack capacity and where to position it. Although uncertainty in demand always requires some buffer, the effects can be mitigated with pooling and supply flexibility [7].

This article is part of a research program aiming to provide insights into using quantitative methods to (re-)design organizations (i.e., partitioning departments and delegating decision authority) with uncertain demands and uncertain throughput times needing short reaction times through an information processing approach. We applied and tested the developed methods with regard to a large perinatology care system. This consisted of three sub-studies.

The first study [10] addressed the need for the care system to be flexible: how to assign the staff (nurses) to where there is work? The perinatology care system has departments that need different types of skilled nurses. Having all nurses be flexible is unrealistic; our method [10] proposes the number of nurses that should be cross-skilled to create the necessary rate of flexibility. However, it is also required that the various departments are managed in an integrated manner to make sure that the patient demand is absorbed, both at the level of departments and in the whole care system. At the same time, the number of transfers of nurses between departments should be restricted. We showed that, in this way, efficient throughput of patients and the need for flexibility is realized and limited.

The second study [11] addressed how the organization structure influences the information processing quantity and its effect on the quality of decision-making for workload control. The quality of decision-making is determined by the time needed for processing and the granularity of the information. An entropy concept can be used to measure the uncertainty associated with specific (organization) structures [12,13,14]. According to Shannon [13], the higher the uncertainty in the system, the higher the entropy; consequently, more information is required to understand what is happening in it. The entropy concept has been applied in various fields, such as statistical thermodynamics, economics, operations research, queueing theory, manufacturing, and many more [12]. Our study showed that entropy is very suitable for measuring the needed information and designing the organization structure, fulfilling the required need for information processing.

While the first study [10] addressed the need to manage various departments in an integrated manner to deal with variable demand, and the second study [11] addressed how to quantify the integration of organization based on the information entropy theory, this current study operationalizes the organization as a queuing network that has to be coordinated. We studied how the total entropy of demand for perinatology care can be partitioned in such a way that the entropy for the expected care of each department is minimized to increase the requisite variety [15] of the queuing network. In terms of the queuing networks, each department *i* becomes a queuing system with a certain arrival rate $\lambda_{i}$ and processing rate $\mu_{i}$. Both the arrival rate and the processing rate have patterns characterized by entropy and variety. The uncertainty that the organization is confronted with during each operational period (i.e., the next day, the day shift, the next hour) for our perinatology care system is determined both by the variety and entropy of demand. The variety concerns the type of care services to be provided, the entropy, and the uncertainty of which services have to be provided for the next hour, shift, or day. Most care services typically belong to a specific department. For others, there is some freedom to allocate them to that department to ensure that a queuing system is most stable. These care services can be handled tactically, where department heads exchange the service provision to stabilize their workload [16]. This exchange process is a type of regulation or organization of the queueing network to improve the requisite variety. The aim of this study is directed to the operational decision-making levels making use of information and information processing theory.

We summarize the research questions as follows:

- *How can we, by decision-making on an operational level, partition the organization in such a way that the entropy is minimized?*
- *How should this decision-making be organized?*

This paper is structured as follows: We discuss how organizations can be considered as queueing networks. Next, we introduce entropy theory to consider queueing network coordination as an information processing problem. We apply these theories and methods to a case study, the perinatology care system, with several decision-making models to optimize the care system as an entropy-reducing organization.

## 2. Theory

### 2.1. Queuing Theory and Utilization in a Hospital System

Servers need capacity. Various capacities are often needed when providing services, e.g., rooms, equipment, and staff. In a perinatology care system, some equipment is not transportable, and rooms can be dedicated. Design and configuration choices can limit operational flexibility. In our study, we assumed that these choices had been made and did not limit the flexible assignment of staff, but it may restrict the assignment of processes to departments. This is realistic, as some perinatology care systems design their delivery rooms in such a way that they can serve as patient rooms or as rooms where both ill women and ill newborns can stay. In our study, we focused only on nurses, as they also represented part of the focus of our two previous studies.

Each department can be considered a queueing system, such as the M/M/c system. In an M/M/c system, patients arrive at a rate (λ) according to a Poisson process and stay in the department according to the service rate (μ). The department has a finite capacity of c, whereas the resource capacity is determined per department far in advance, based on the forecasted demand. Based on the available capacity (c), patients are admitted on a first-come, first-served basis. Examples of measured performances include the average time a patient spends in the system, server utilization, and the number of patients in the system at time n.

Interrelated queuing systems can be analyzed as a queuing network, where each routing path connects each department. Given the route probability of patients ($r_{ij}$) from ward i to ward j and $\lambda_{i}$ as the arrival rate of patients to department i, the effective arrival rate (*Eff* $\lambda_{i}$) is the external arrival (if any) to ward i, and the internal patients that finished their care at ward j are then routed to ward i for the next stage of care services.

(1) $$Eff\;\lambda_{i}=\lambda_{oi}+\sum_{j=1}^{M}\lambda_{j}r_{ji}\;$$

The utilization of wards per day is calculated based on the following traffic equation:

(2) $$\rho =\frac{\lambda }{c\cdot \mu }$$

The utilization formula (ρ=λ/cμ) from the queuing theory intuitively portrays the following: if patients (λ) are arriving faster than they can be served (capacity *c* and the service rate μ), then utilization will be more than 100%. Consequently, the queue will grow, and the system will become unstable. Thus, for a stable system, a department requires a condition where λ<cμ.

Although the capacity c is determined in advance, dependent on the workload of departments, if needed, possible nurses can be temporarily assigned to another department. Each department can be in a state of high, medium, or low workload in an operational time horizon (a day, a shift, an hour). A department can switch staff if one department is in a high-workload state and another in a low-workload state [16]. For instance, if we have a system with four departments, each department has queueing system $M/M/c_{i}$. If a nurse is reassigned from department 1 to, for example, department 2 for a certain period (e.g., the next two hours), the capacity of the following two queueing systems changes: $c_{1}-1,c_{2}+1$. M/M/ does not change in this process. If the variety of services changes, the M/M/ of at least two departments’ changes.

Stability of each department and the whole care system is needed. How can this be accomplished? The variety and its entropy should be reduced as much as possible, but still, the transfer of nurses is sometimes needed. As stated earlier, a stable queueing system should have a utilization degree of less than 100%. To avoid peaks, the average utilization degree could be set (as an example) to 80%. This has to be determined through experimentation. At the same time, the care system would like to restrict the number of transfers between departments.

We propose the following two methods: Portfolio optimization [17] and the efficient coordination of workload and the reallocation of nurses from an information processing perspective [18]. In terms of portfolio optimization, Van Merode et al. [9] suggested considering the correlation perspective when calculating the required capacity, whereas departments’ demands that correlated negatively were clustered together. This is in line with the portfolio approach of Markowitz [17]; an optimal portfolio is chosen when the variance in the workload of the shop is less than the sum of variances of the several processes (i.e., demand) allocated to the shop. In statistics, a perfect negative correlation is represented by the value −1, and +1 indicates a perfect positive correlation. For example, if units *i* and *j* have a negative correlation, *i* will decrease as *j* increases in value; similarly, if *i* decreases in value, *j* will increase. Any value closer to 0 is considered a weakly negative or positive correlation. Hence, the theory [17] suggests that the less correlated each demand is with one another, the less volatile the portfolio (of the shop) will be [18]. A recent study applied fictitious data to illustrate the theoretical value of using a portfolio approach in a hospital capacity pooling context and showed some opportunities, such as increased service levels, given the same capacity [19]. When using this approach to structure the organization, one also reduces the uncertainty of patient demand associated with the departments. In other words, this approach tries to optimize uncertainty reduction by organization structuring.

As for determining the coordination system, we must first know the coordination task. In our approach, sufficient nurses in a department determine the flow. If there are not enough nurses, this should be signaled, and a nurse is reassigned upon receiving the signal. However, by whom and to whom? And when a nurse is transferred to a department, how long will the nurse stay there? This is considered an information processing task and is discussed in the following subsection.

### 2.2. Information Processing Organizations in Hospitals

According to Ashby’s law of requisite variety [15,20], a system can be classified into the following three stages: an input (i.e., that has a variety and uncertainty), a regulatory process, and an outcome. A regulatory process responds to the input variation, which in turn, leads to the desired output. In other words, given the uncertainty that can disturb the organization in various ways, the organization needs multiple options to solve it.

In the context of mismatches between the available nurses and the actual demand in real-time, certain actions are needed to solve the mismatches, such as reallocating nurses from an under-utilized ward to the needed ward or admitting patients to an under-utilized ward. The following several scenarios are possible when a nurse is reallocated to a department with a high workload:

- Once the workload is back to a medium or low level, the nurse returns to his/her primary department;
- Once the workload is at a medium or low level, the nurse has to ask the care system’s management what to do: stay, return to the home department, or go to another department;
- The nurse stays until the end of a period, and then he/she returns to the primary department;
- The nurse stays until the end of a period and asks the care system’s management where to go;
- The nurse stays until the end of their shift.

To organize the above scenarios in practice, three essential elements play a role:

- The distribution of authority

Following the framework of Sandoe [21], processing the information to coordinate the workload and reallocation of nurses in real-time can be carried out through *hierarchy*, *network*, and *hub* strategies. For the *hierarchy* strategy, the decision-making authority to allow nurses to go or return to a specific department is positioned higher in the organization pyramid, for example, at the manager level. Local information regarding the workload is centralized and processed by the decision-maker (i.e., the manager). She/he provides the solution to reallocate nurses to a specific department.

In the *network* strategy, the decision authority to reallocate nurses is given to the staff at the operational level, such as the planner. Each planner contacts other planners to obtain and share the needed information. In this strategy, the local information regarding the workload is decentralized at the planner of each unit. In the *hub* strategy, the decision-making authority is also given to the planner. However, the strategy allows each planner to organize the process with the help of centralized information technology. Each planner is responsible for managing the local information available in the information system that all the other planners can assess. Contrary to the *network* strategy, where the information is decentralized at each planner, the *hub* strategy centralizes the detailed information with the help of information technology. Nevertheless, each planner still decides what (detailed) information is essential and acts accordingly.

- The measurement of workload

Information, such as the expected workload in the coming period, is needed to coordinate the transfer of nurses in the care system. One important piece of information is positional information [11,12], which refers to a patient’s actual position in the system.

- The frequency of capacity adjustments (i.e., per specific planning period or per shift).

The determination of the planning period is also essential. The planning period can be defined as a minimum of a few hours to a maximum of one day, depending on the department’s ability to mitigate capacity problems when understaffing occurs (e.g., by using flexible nurses); however, it also depends on the capability of organizations to process the required information. Among the three strategies discussed by Sandoe [21], information technology is assumed to be available to support information acquisition and processing in organizations, although the level of information aggregation can be varied per organization.

The speed of decision-making in real-time also depends on the level of information aggregation set up by the system. The level of information aggregation involves the concept of coarse-graining. A fine-grained description is a detailed description of the system’s microscopic behavior, while a coarse-grained description is one where the fine detail has been smoothed over through aggregation. The level of information aggregation can further dictate the required frequency of nurse reallocations. When the information from all of the units is aggregated only to report their workload (i.e., the number of occupied beds), instead of every detail on patient mismatches, the department can have a shorter planning horizon (i.e., greater feedback frequency) to acquire and process information from the units.

Furthermore, the organization of local and system-wide information also dictates the speed of decision-making. In the *hierarchy* strategy, local information is positioned locally at each unit (i.e., decentralized). Local information is microscopic. When the decision-maker needs to execute a task to solve the supply and demand mismatches, she/he needs system-wide information to avoid making suboptimal interventions. She/he then integrates the local information to obtain the aggregated system information. In the *network* strategy, the local information is decentralized at each unit. However, information aggregation does not necessarily happen because the decision-maker (i.e., the planner) decides which local information from which unit is needed to complete the tasks. For the *hub* strategy, the decentralized local information is automatically integrated. Based on this, the decision-makers execute their task.

### 2.3. Measuring Entropy in Hospital’s Information Processing

Due to the uncertainty of patient demand prominent in hospital operations, developing capacity planning for short-term planning is essential in developing real-time control of hospital resources. The literature suggests that organizations need to build information processing capability to minimize decision-making delays in organizations [12,22,23]. A given system design follows a specific structure in which information is gathered and processed. An information entropy model can be used to measure the amount of information [14]. Entropy is defined as a measure of randomness or disorder in a system [12,24]. Information entropy values can be assigned to specific structures so that we can compare them to gain insights into the factors that lead to effective decision-making. As the higher the uncertainty in the system, the higher the entropy; consequently, more information is required to understand what is happening in it. Processing too much information will lead to a delay in decision-making.

To calculate the information entropy (H), we can follow Shannon’s information entropy formula [13]. If the average uncertainty associated with an outcome is represented by a discrete random variable *X*, the Shannon’s information entropy of a discrete random variable of X with *n* outcomes ($x_{1},x_{2},\;\ldots \;x_{n}$) is as follows:

(3) $$H\;\left(x\right)=-\sum_{i=1}^{n}P\left(x_{i}\right)\log_{2}P\left(x_{i}\right)$$

Probabilities are needed to calculate entropy, which depends on the available information in the system. When such information is unknown, assumptions are made to estimate probability. Instead of using an estimation, we can consider the extremum of information entropy [12]. The extremum of information entropy is used to discover the probability distribution, leading to the highest value for this uncertainty, assuring that no information is carelessly assumed.

In this study, we used entropy theory to measure the three types of uncertainty that a unit may experience; the uncertainty of patients’ arrival into the care system (i.e., the arrival uncertainty), the uncertainty of patients’ actual position within the care system (i.e., the position uncertainty), and the uncertainty of (detailed) information needed to solve the supply–demand mismatches (i.e., the decision-making uncertainty).

- Arrival entropy

In the queuing network, the effective arrival rate (*Eff* $\lambda_{i}$) is the external arrival (if any) to department i and the internal patients who have finished receiving their care at ward j and are then routed to ward i for the next stage of care services, as given in Formula (1).

The relative demand d of patients who have arrived in department i is

(4) $$d_{i}=\frac{Eff\lambda_{i}}{\sum_{i=1}^{N}Eff\lambda_{i}}$$

When N is the number of departments, the arrival entropy of all departments in the system$\;H_{as}$ is

(5) $$H_{as}=-\sum_{i=1}^{N}\left(d_{i}{log}_{2}d_{i}\right)$$

To calculate the scenario of maximum entropy, the maximum probability of patients who have arrived in department i is

(6) $$d_{i}^{max}=\frac{1}{N}$$

The maximum arrival entropy of all departments in the system ($H_{as}^{max})$ is

(7) $$H_{as}^{max}=-\sum_{i=1}^{N}\left(\;d_{i}^{max}{log}_{2}d_{i}^{max}\right)$$

- Positional Entropy

When a hospital system is characterized as having several workstations (i.e., departments) connected in a network, uncertainty regarding patients’ actual positions in the system can cause delays in the decision-making process. We refer to this as positional uncertainty [12]. This information is needed for departments to calculate the expected workload. When positional uncertainty is high, the expected workload may be less accurate; hence, mismatch occurrences are still possible.

Positional uncertainty can be measured with information entropy. Given a specific system structure, we need the information of $P_{ki}$; that is, the probability of a patient k(k=1,2,…,K) progressing to department i(i=1,2,…,N). For ∀k, $\sum_{i}P_{ki}=1.$ Having $P_{ki}$, the positional entropy of a patient in the system ($H_{k})\;$can be calculated as

(8) $$H_{k}=-\sum_{i=1}^{N}\left(P_{ki}\log_{2}P_{ki}\right),\;\forall k$$

The positional entropy of all patients in the system ${(H}_{ps})$ is

(9) $$H_{ps}=-\sum_{k=1}^{K}\sum_{i=1}^{N}\left(P_{ki}\log_{2}P_{ki}\right)$$

- Decision Structure Entropy

When a hospital system is characterized as having several layers of decision functions, positioned vertically or horizontally, as discussed, following the framework of Sandoe [21], uncertainty concerning the mismatches in detail causes delays in real-time decision-making. We applied the same concept as in Formula (3). Given a specific system structure, $P_{ab}$ is the probability of information being processed by decision maker a(a=1,2,…,A), given the required information from source b(b=1,2,…,B). For ∀a, $\sum_{b}P_{ab}=1.$ Having $P_{ab}$, the decision structure entropy for position a can be calculated as follows:

(10) $$H_{a}=-\sum_{b=1}^{B}\left(P_{ab}\log_{2}P_{ab}\right)$$

The decision structure in the system is ($H_{ds}$):

(11) $$H_{ds}=-\sum_{a=1}^{A}\sum_{b=1}^{B}\left(P_{ab}\log_{2}P_{ab}\right)$$

Intuitively, when the organization as a whole loses positions, the entropy is reduced, and when it gains positions, the entropy increases [24].

- The total associated entropy in the system

For each care system configuration and associated coordination system, we measure the uncertainty based on the arrival uncertainty, the patient position uncertainty, and the decision-making uncertainty. Since we quantify the uncertainty in terms of the entropy in bits, the total uncertainty in the system is the total entropy of the three entropy measures.

(12) $$Entropy\;total=H_{as}+H_{ps}+H_{ds}$$

## 3. Methodology

### 3.1. Study Settings

**Figure 1:**
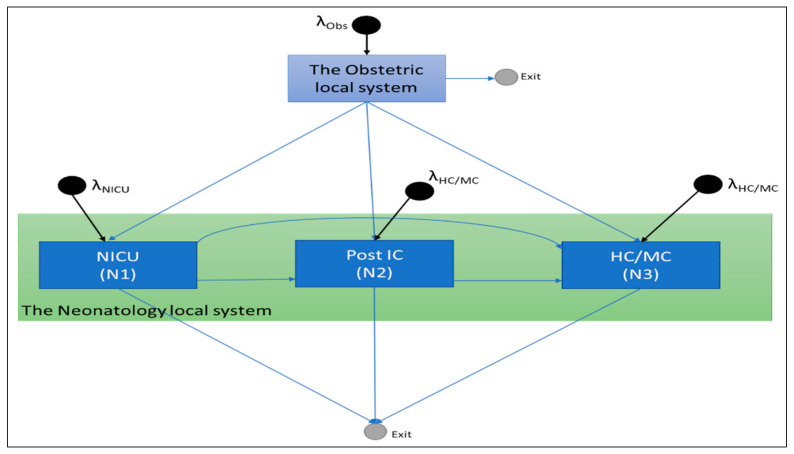
The flow of newborns in the perinatology care system in Radboud UMC.

At Radboud University Medical Center in Nijmegen, the Netherlands, the perinatology care system consists of two local systems: obstetrics and neonatology. The obstetrics local system has three units: the nursery ward (25 beds for adults and 7 for newborns), the obstetrics high-care unit (OHC) (3 beds), and the delivery room (6 beds). These different units are based on the type of care given to the adults (the mothers) and will hereafter be referred to as O1, O2, and O3, respectively. There are also the following three units within the neonatology local system: the neonatal intensive-care unit (NICU) (14 beds), the neonatal post-intensive-care unit (Post-IC) (4 beds), and the neonatal high-care/medium-care unit (HC/MC) (11 beds). We will refer to these units as N1, N2, and N3, respectively. There are a total of 70 beds in the perinatology care system. In this study, we focused on the flow of newborns in the perinatology care system, as shown in Figure 1.

### 3.2. Study Method

**Figure 2:**
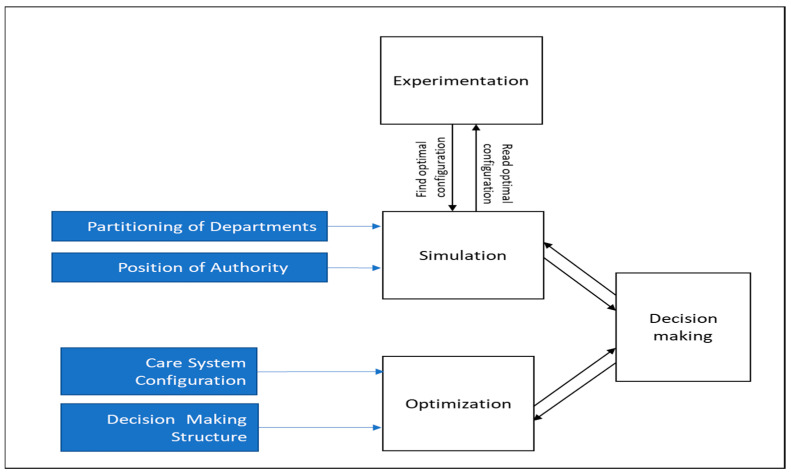
Simulation and experimentation flow chart.

The method of this study is presented in Figure 2. We begin by accepting the current perinatology configuration (i.e., our case study for the care system) with 2 separate departments with 6 units total, whereas 4 units provide care for newborns. This is decided at a strategic level, with a planning horizon of 1–2 years (or possibly longer). At this planning level, the decision-making structure is also defined, following the framework of Sandoe [21] (i.e., the *hierarchy* strategy).

Given the information presented in Figure 1, we developed a queuing network model in EXCEL. Discrete Event Simulation (DES) was performed to evaluate the effect of variable demand on a daily level on the perinatology case system’s care configuration and decision-making structure.

In this study, we experimented with different configurations of department partitioning, modeled as an M/M/c queuing network. We developed four scenarios of department partitioning; Model 1 is the current situation with 5 separated departments, Model 2 is the combined settings of the high- and medium-care unit (N3) with the nursery ward (O1), Model 3 is the combined settings of the high- and medium-care unit (N3) with the post-intensive-care unit (N2), and Model 4 is the combined settings of the post-intensive-care (N2) unit with the nursery ward (O1). The overview of variables is given in Table 1.

Given the simulated input data of patient arrivals, the associated length of stay, and how departments are partitioned, we observe the expected days the system needs to reallocate nurses. The steps are presented in Algorithm 1. Output variables are R1, R7, R13, and R19. Furthermore, we observe the effect of department partitioning on the effective arrival uncertainty (i.e., R2, R8, R14, and R20) associated with the structure. The steps are presented in Algorithm 2. We then observe the patients’ positional uncertainty (i.e., R3, R9, R15, and R21) when making real-time decisions. The steps are presented in Algorithm 3. Finally, we model the decision-making structures of the perinatology care system following the framework of Sandoe [21] (i.e., *hierarchy*, *network*, and *hub*) and emphasize where the decision power is positioned. The steps are presented in Algorithm 4. Based on the experiment results, we found the optimal care configuration and decision-making structure, which is the structure that results in minimum supply–demand mismatches and minimizes associated entropies.
**Algorithm 1: Calculating the number of days with overutilized staffed beds**1.Construct the queuing network of departments *i =* {1, 2, 3, …, *N*}, for example as {Delivery room, NICU, Post-IC, HC/MC, Nursery ward}, based on the care configuration of Model 1.
Define the probability route $r_{ij}$ of patients moving from department i to j, i≠j based on the historical data.Assign the number of staffed beds $c_{i}$ ∀iAssign the expected length of stay $\mu_{i}$ ∀i
2.Run a simulation with varying arrival rate $\lambda_{i}$ of ∀i for *d* number of days. In this study, S={1, 2, 3, …d}, with $\left|S\right|=365$.
∀i and ∀S, calculate the effective arrival rate of patients $Eff\;\lambda_{i}^{S}$ based on Formula (1)∀i and ∀S, calculate the utilization rate $\rho_{i}^{S}$ of based on Formula (2)∀i and ∀S, calculate the utilization $U_{i}^{S}$ = $\rho_{i}^{S}\times c_{i}^{S}$∀i and ∀S, calculate the overutilization and underutilization
$\mathrm{If}\;U_{i}^{S}>c_{i}^{S}$, department i$\;\mathrm{has}\;U_{i}^{S}-c_{i}^{S}$ overutilized beds.$\mathrm{If}\;U_{i}^{S}<c_{i}^{S}$, department i$\;\mathrm{has}\;c_{i}^{S}-U_{i}^{S}$ underutilized beds
∀S, calculate the overutilization and underutilization of the care system
Overutilized system ($U_{over}^{S})$ is $\sum_{i=1}^{N}(U_{i}^{S}-c_{i}^{S})$, ∀SUnderutilized system ($U_{under}^{S})$ is $\sum_{i=1}^{N}(c_{i}^{S}-U_{i}^{S})$, ∀S
3.For the care system:
Calculate the average number of overutilized beds per day $({\bar{U}}_{over}$) with $\frac{\sum_{S=1}^{d}\left(U_{over}^{S}\right)}{d}$Calculate the average number of underutilized beds per day $({\bar{U}}_{under}$) with $\frac{\sum_{S=1}^{d}\left(U_{under}^{S}\right)}{d}$∀S, we can calculate the frequency of d with $\left(U_{over}^{S}\right)$ as the days in which we need to reallocate nurses.
4.Construct the queuing network of departments {1, 2, 3, …, *N*} based on the care configuration of Model 2, 3, and 4 by repeating steps 1a–3c for each model.


**Algorithm 2: Calculating the arrival entropy**

Construct the queuing network of departments *i* = {1, 2, 3, …, *N*}, based on the care configuration given in Model 1.Construct the probability route $r_{ij}$ of patients moving from department i to j based on the care configuration.Given the historical data, calculate the arrival rate $(\lambda_{i}$) of patients to department i. Then calculate the effective arrival rate department $(Eff{\;\lambda }_{i}$) based on Formula (1).Calculate the total arrival of the system $\sum_{i=1}^{N}\left(Eff{\;\lambda }_{i}\right)$Calculate the relative arrival demand of each department *i* with Formula (4). Calculate the arrival entropy of all departments in the system with Formula (5).Calculate the relative arrival demand with the maximum probability distribution with Formula (6), and the maximum arrival entropy of all departments in the system with Formula (7).Repeat steps 1–6 for each Model 2, 3, and 4.



**Algorithm 3: Calculating the positional entropy**

Construct the queuing network of departments *i* = {1, 2, 3, …, *N*}, based on the care configuration given in Model 1.Construct the probability route $r_{ij}$ of patients moving from department i to j based on the care configuration. Calculate $P_{ki}$; that is, the probability of a patient k={1,2,…,K} progressing to department i(i=1,2,…,N). For ∀k, $\sum_{i}P_{ki}=1.$$\mathrm{Having}\;\mathrm{obtained}\;\mathrm{the}\;P_{ki}$, calculate the positional entropy of a patient in the system with Formula (8). Calculate the positional entropy of all patients in the system with Formula (9).$\mathrm{Having}\;\mathrm{obtained}\;\mathrm{the}\;\mathrm{maximum}\;\mathrm{probability}\;\mathrm{distribution}\;\mathrm{of}\;P_{ki}=1/N$, calculate the maximum positional entropy of a patient in the system with Formula (8). Calculate the maximum positional entropy of all patients in the system with Formula (9).Repeat steps 1–4 for Models 2, 3, and 4.



**Algorithm 4: Calculating the decision-making entropy**

Construct the coordination system, Model 1, based on the framework of Sandoe (1998) [21], namely, *hierarchy*, *network,* and *hub*, with several information points of decision-makers a={1,2,…,A}, such as {manager, team manager, planner/team leader, nurses}. And the source of information b={1,2,…,B}, such as {team manager, planner/team leader, nurses}.Construct $P_{ab}$, as the maximum probability of information being processed by decision maker a={1,2,…,A}, given the required information from source b={1,2,…,B}. ∀a, $\sum_{b}P_{ab}=1.$ Having obtained $P_{ab}$, calculate the decision structure entropy in position b with Formula (10) and in the system with Formula (11).Repeat steps 1–2 for Models 2, 3, and 4.


## 4. Results

### 4.1. Analysis of Care System Configuration

**Figure 3:**
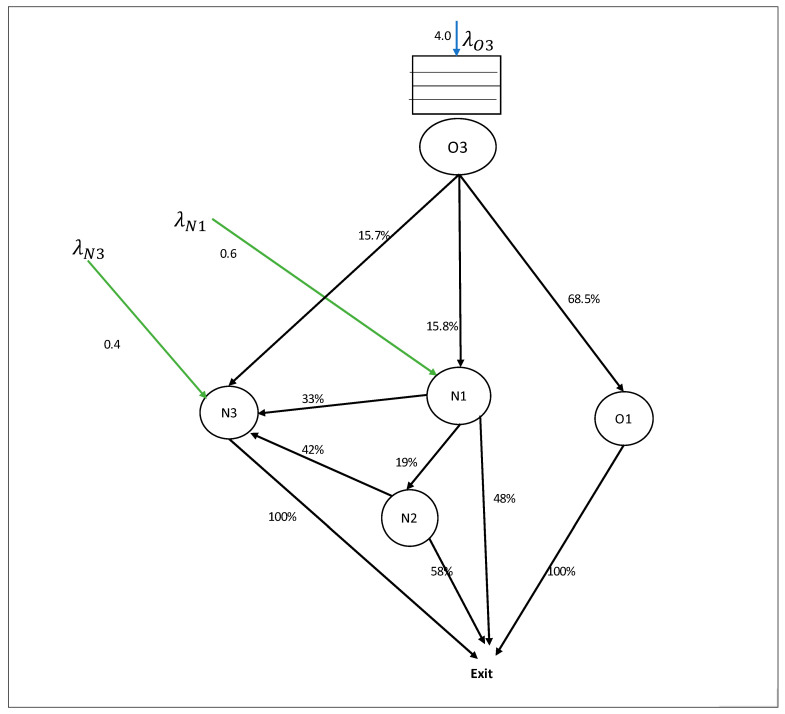
Newborn flows in the perinatology care system, where the patient movement % between units was calculated based on historical data of patient movements from unit *i* to unit *j*. The average birth per day in the delivery room (O3) is four newborns. In total, 68.5% of the newborns were healthy and transported to the obstetric local system’s nursery ward (O1). A total of 15.8% of the newborns need intensive care services at the NICU (N1), and 15.7% need high-care/medium-care services at the HC/MC Unit (N3). Besides newborn arriving through the O3, NICU (N1) and HC/MC Unit (N3) also admitted newborns from other hospitals. The average external arrival (from other hospitals) for the NICU (N1) is 0.6 newborn/day, and 0.4 newborn/day for the HC/MC Unit (N2).

As stated earlier, we focus on the flow of newborns in the perinatology care system, as given in Figure 1. Specifically, this includes the neonatal intensive care unit (N1), the neonatal post-intensive-care unit (N2), and the neonatal high-care/medium-care HC/MC unit (N3) from the neonatology local system. And it also involves the delivery room (O3) and the nursery ward (O1) from the obstetrics local system. Based on the performed data analysis for the data of the year 2018, the flow of patients in the care system is given in Figure 3.

The performance of the current care configuration is presented in Table 2. Neonatal post-IC was the bottleneck, with the highest capacity utilization in the system. HC/MC of the neonatology department has a utilization below 50%, similar to the nursery wards of the obstetric department. While the HC/MC cares for sick newborns, the nursery wards care for healthy newborns.

For the perinatology care system, we can generally refer to O3 as the upstream unit, N1 as the intermediate unit, and N2, N3, and O1 as the downstream units. The downstream units need a certain buffer capacity to deal with uncertainties in terms of patient arrivals in the system. Pooling capacities to deal with uncertainties can be realized by combining the downstream units. Analysis of correlations performed towards the downstream units is given below. As is shown in Table 3, the combination of wards with a negative correlation is found only in the nursery wards (O1) and the post-IC unit (N2). However, it is considered to be a weak negative correlation as the value is closer to zero. Similarly, the post-IC unit (N2) and the high-care/medium-care unit (N3) have a weak positive correlation. This means that, when combining N2 with either O1 or N3, as the workload of N2 increases, the workload of N3 and O1 are not simultaneously increased.

### 4.2. Simulation

#### 4.2.1. Supply–Demand Mismatches

**Figure 4:**
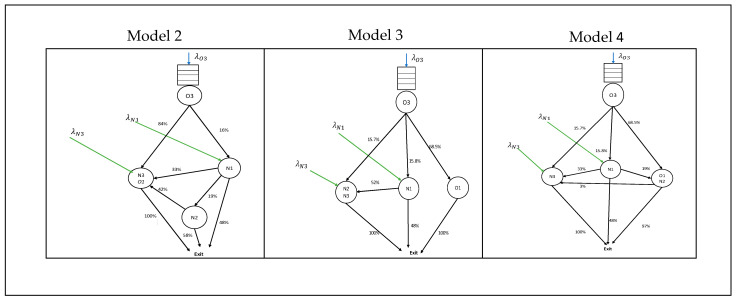
Flows of newborns for Model 2, Model 3, and Model 4. Combining the capacity of two units means combining the arrival demand associated with the unit. The new patient movement % between units is calculated based on historical data of 2018.

At the operational level (i.e., closer to the actual working time), the patient demand might differ on a unit level compared to the predicted result. We then simulated the variable arrival demand on the current and the new care configurations, as shown in Table 1. The flow of patients for Models 2, 3, and 4 used for the simulation is given in Figure 4.

We evaluated the supply–demand mismatches based on the average number of over/underutilized staffed beds, and the number of days with overutilized staffed beds, following the steps given in Algorithm 1. As is shown in Table 4, the number of days with overutilized staffed beds is decreased when combining N2 with either O1 or N3, indicating a more system-wide balance in the care system when dealing with the external uncertainty of patient demand in real-time. The models with negatively correlated demand resulted in the lowest average overutilized beds/wards (Model 4).

#### 4.2.2. Arrival Entropy

To manage the arrival uncertainty, departments may be clustered or partitioned. The current configuration has five varieties {delivery room, NICU, post-IC unit, HC/MC unit, nursery ward}. When combining any of the downstream units, the variety decreases; hence, the entropy decreases, as shown in Table 5. Given the historical data, pooling the HC/MC unit (N3) with nursery wards (O1) seems to be able to absorb the arrival uncertainties the most. Assuming maximum entropy, pooling any downstream departments minimizes the arrival entropy.

#### 4.2.3. Positional Entropy

Given the prior decision concerning the configuration of departments (i.e., Model 1 to Model 4), decisions still need to be made in real-time to allocate patients to the right unit within the system. As is shown in Table 6, pooling any downstream departments minimizes the positional entropy. The structure that minimizes the positional entropy the most is when the post-IC unit (N2) is pooled with the HC/MC Unit (N3), resulting in 2.2 bits, given the historical data, and 2.58 bits when assuming maximum entropy.

#### 4.2.4. Decision-Making Structure

In the current system, the number of nurses scheduled per day is given in Table 7. This is based on the nurse-to-patient (NtP) ratio, derived from expert opinions and practical justifications, which have evolved from the past. When supply–demand mismatches are experienced in real-time, such as when department(s) experience an overutilized staffed bed, the decision-maker needs to decide on the capacity adjustment. For example, given a certain planning period (e.g., per shift), reallocating nurses to a department with an overutilized staffed bed.

**Figure 5:**
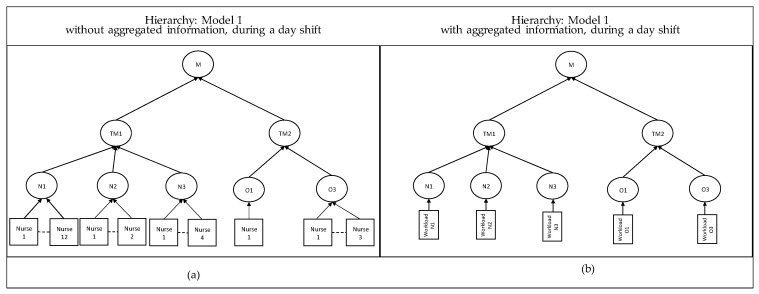
Hierarchy decision-making structure for Models 1, at the day shift. (**a**) portrays the system with detailed information decentralized at the nurse level, while (**b**) portrays the system with aggregated information. When units are combined, the nurses from the combined department report to the assigned team manager, depending on the structure given in Models 2, 3, and 4.

In Figure 5, the *hierarchy* system, the decision power is positioned at the manager level, with a system-wide overview. Once the workload is medium or low, the nurse has to ask the team managers what to do as follows: stay, return to the home department, or go to another department. Alternatively, the nurse stays until the end of a period and asks the team managers where to go. The team managers gather the needed local information from all nurses from each unit of his/her departments (Figure 5a) or gather the aggregated information that only report the expected workload for each unit (Figure 5b) and report it to the manager for approval.

**Figure 6:**
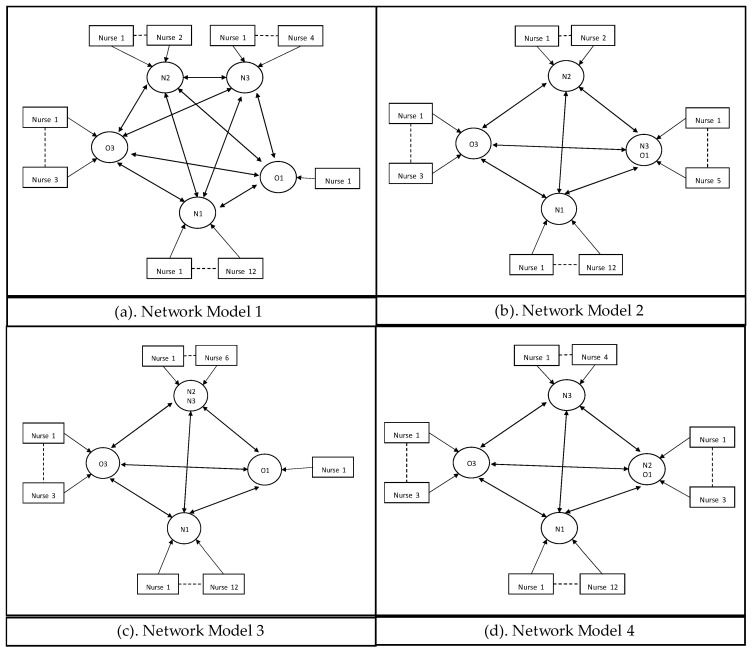
Network decision-making structure for Model 1 (**a**), Model 2 (**b**), Model 3 (**c**), and Model 4 (**d**).

In Figure 6, the *network* strategy, the decision-making power is positioned lower at the operational level, the planners, or the team leaders of each unit. When mismatches occur at their unit, given the local information, they contact the other planners (or the team leaders) from other units individually to reallocate nurses in the system.

**Figure 7:**
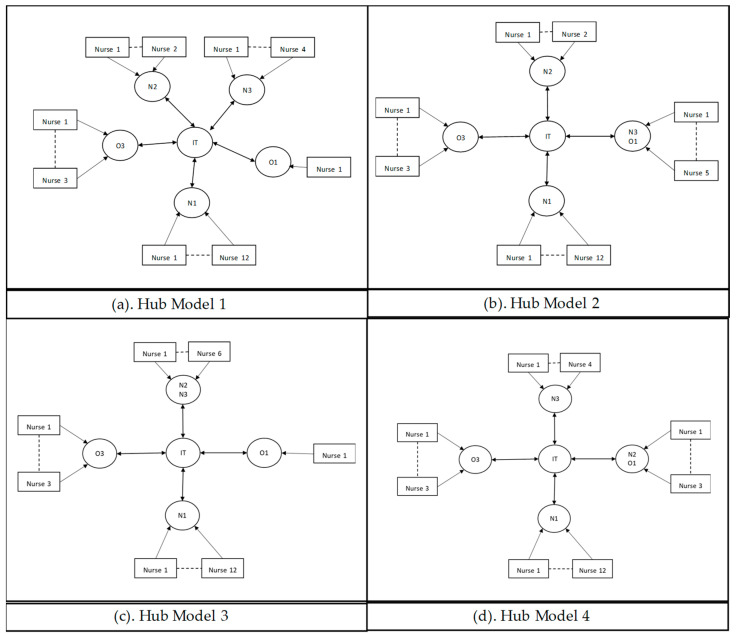
Hub decision-making structure for Model 1 (**a**), Model 2 (**b**), Model 3 (**c**), and Model 4 (**d**).

In the *hub* strategy, as given in Figure 7, decision-making power is also positioned lower at the operational level; the planners or the team leaders of each unit. They have access to a centralized information system that holds system-wide local information (i.e., each unit’s demand and capacity information) so that each decision-maker has sufficient information to reallocate nurses.

The results of the entropy analysis are given in Table 8. When the decision-making requires local information, the structure with the lowest associated uncertainty is the *hub* strategy, where the planners have the power to decide where the nurses should be reallocated within the system. Moreover, given a certain planning period (e.g., per shift) and the expected workload at each local department, planners decide whether the reallocated nurses should stay at the current department or return to their home department. These planners are connected through centralized information technology, with integrated detailed information from other departments influencing the speed of information gathering and processing. Each planner still decides what (detailed) information is essential and acts accordingly.

In Table 9, entropy analysis is given when the information needed for decision-making is aggregated only to report a workload per unit instead of per nurse. Compared to the results in Table 8, the associated uncertainty is decreased, hence the time needed to acquire and process information for decision-making. As can be seen, although the *hub* strategy provides the lowest associated entropy, the *hierarchy* structure is the next best strategy.

## 5. Discussion

In this study, we portray how organizations can be considered queueing networks and apply the entropy theory to interpret the queueing network coordination as an information processing task. We answer and discuss our research questions within the following.

### 5.1. How Can We, by Decision-Making on an Operational Level, Partition the Organization in Such a Way That the Entropy Is Minimized?

For a hospital system, the downstream departments need a certain slack capacity to deal with the uncertainty of patient arrivals. The analysis of the current situation in the perinatology care system showed that the neonatal post-IC unit (N2) was the bottleneck, with the highest capacity utilization in the system. When considering the correlation perspective, we investigated the pooling of the HC/MC unit (N3) and nursery ward (O3), the pooling of the post-IC unit (N2) and HC/MC unit (N3), and the pooling of post-IC unit (N2) and nursery ward (O3). The analysis of correlations showed that combining N2 with either O1 (Model 4) or N3 (Model 3) would be best; as the workload of N2 increases, the workload of N3 and O1 tend not to increase simultaneously, balancing out the system utilization when dealing with the uncertainty of patient arrivals.

In the current system, the one with the negative correlation is the post-IC unit (N2) of the neonatology care system and the nursery ward (O1) of the obstetric care system. This can happen due to the different types of patients allocated to the unit. For instance, the post-IC unit (N2) is designed to care for sick newborns, while the nursery ward (O1) is intended to care for healthy newborns. The nurses’ specialization focus also differs for these units; the neonatology nurse is assigned to N2, and the obstetric nurse is assigned to O1. In other words, these two departments have different systems. Given the different specialization focus of these units (post-IC unit—N2, with the nursery ward—O1), implementation might be challenging. However, as we previously studied in Winasti et al. [10], the effects of pooling can be achieved with a small amount of flexibility. Creating a small percentage of nurse flexibility between the post-IC unit and the wards is a contender strategy to deal with uncertain demand in real-time.

**Figure 8:**
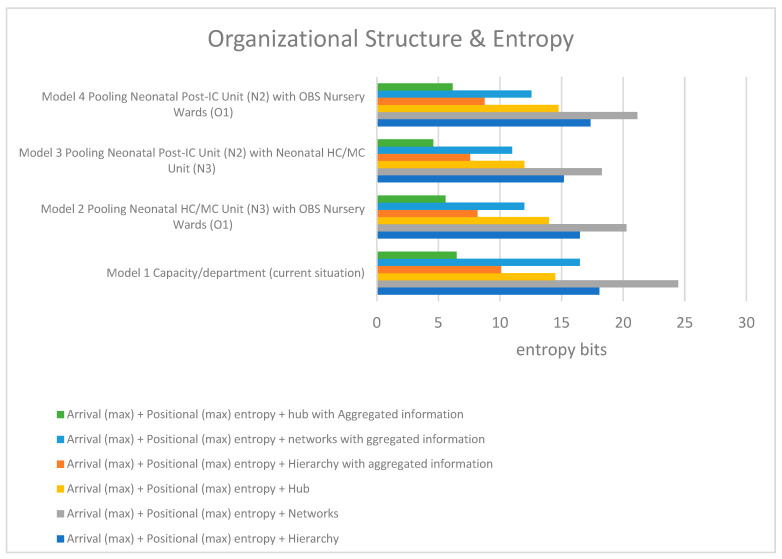
The overview of entropy analysis.

Furthermore, we used entropy theory to measure the following three types of uncertainty a unit may experience: arrival, position, and decision-making uncertainty. The total uncertainty associated with the model and decision-making structure is the summation of arrival, position, and decision-making entropies, as shown in Table 10 and Figure 8. When making a decision regarding the design that can minimize uncertainty, pooling the HC/MC unit and the post-IC unit (Model 3) with a strategy where the decision power is positioned at the operation level and with the help of a centralized information system is the best, with or without the aggregation of information.

### 5.2. How Should the Decision-Making on an Operational Level Be Organized?

The speed of gathering and processing the needed information influences the effectiveness of the decision-making process at the operational level. First, the organization of local and system-wide information dictates the speed of decision-making. When the local information is integrated, the decision power is better positioned at the operational level of care activity (i.e., the *hub* strategy), as indicated by the entropy analysis. This is aligned with a recent study by van der Ham et al. [25], where they observed that decisional support by producing data and information for operational decision-making seems to work best when provided to the operational decision-makers, compared to when it is provided to (tactical and strategic) manager(s).

When the local information is not integrated to support the system-wide (i.e., horizontal) overview, hospitals can better use the *hierarchy* model, where the manager holds the decision-making power with system-wide information. Complementary to the *hierarchy* strategy, hospitals can invest in a vertical information system (VIS) to allow speedy information exchanges [26] between the planners, team managers, and the manager of the care system.

Second, the speed of decision-making in real-time also depends on the level of information aggregation set up by the system. The manager should make use of aggregated information (e.g., workload with the number of understaffed or overstaffed beds) instead of detailed information to solve supply–demand mismatches at the operational level. As can be seen in Table 10, the *hierarchy* model with the aggregated information performed better than the *hub* strategy without the aggregated information. These results provide insights into the role of aggregated information in optimizing decision-making process in hospital operations. As we used the workload (i.e., under/overstaffed beds) as the aggregated information, further research is needed to specify other relevant information that can be used for managing hospital operations.

## 6. Conclusions

### 6.1. Practical Implications

Organization structuring or ‘to pool or not to pool’ has been the topic of many studies and is still relevant. In this study, we used the correlation perspective as one parameter to combine departments and subsequently evaluate the uncertainties associated with the system. Combining the correlation perspective and the entropy theory is a way to quantify how organizations can be (re)designed.

The design choice to reduce the effect of uncertainty on the organization segment would concern which demand (and its accompanying uncertainty) should be combined. Following Markowitz’s portfolio theory, we combined the departments with less-well-correlated demand. As expected, pooling less-well-correlated wards can lower the supply-demand mismatches portrayed by fewer days with overutilized beds, agreeing with the theoretical results from Fagefors and Lantz [19]. Given the fewer days of mismatches, the frequency of reallocating nurses within the system has also decreased. When the reallocation frequency is decreased, the system is considered to be more stable.

This method can also be used to evaluate which departments should be physically positioned to organize the reallocation of nurses in practice. For instance, positioning respiratory wards on the same floor (or proximity) with neurological wards might benefit the pooling effect. Since respiratory demand has seasonality patterns [27], neurological demand with more acute admissions does not. Combining respiratory demand with another seasonal demand (e.g., cardiology demand, as analyzed in [28]) might not improve the system’s performance.

Furthermore, we showed that pooling departments also decreased the associated uncertainty experienced by the system. Minimizing the arrival entropy means that the system would have fewer expected mismatches in real-time. Minimizing the positional entropy means the system would have a more accurate expected workload, leading to more accurate resource planning. Furthermore, minimizing the decision-making entropy means the system would have a speedy decision-making process. The care system can opt for the configuration that provides the lowest total entropy by quantifying the three entropies.

### 6.2. Limitations and Future Research

The current study is not without its limitations. First, implementing such a system must be seen as an evolutionary process. As in complex systems, evolutionary systems work based on ongoing, continuous internal processes of exploration and experimentation. Additional research regarding how the planning system can fit into an evolutionary process is a relevant topic for future study. Second, our proposed method is designed for specific settings of the perinatology care system in Radboud UMC. The interpretations might be limited to the population characteristics that belong to our case study. However, our method provides guidelines for its use in other settings, although variation would be needed when implementing it.

Finally, we stated several times that one of the essential elements for a successful implementation design is adequate vertical and horizontal (i.e., system-wide) information and communication technology (ICT). Empirical research on the decision support system (DSS)’s role in nurse planning and control at the real-time planning level is becoming more important. One example is using a nurse staffing tool alongside predictive analytics that provides actual staffing ratios across outpatient centers [29]. How the staffing tool reflects on the over/-understaffed occurrence is an interesting research direction.

## Figures and Tables

**Table 1:** Overview of variables.

Care System Configuration	Controllable Variable (Name of Intervention)	Dependent Variable
Model 1: Current configuration (five separate units)	Queuing network analysis (i.e., under/overutilized capacity and number of days with overutilized capacity)	R1
	Arrival (max) entropy	R2
	Positional (max) entropy	R3
	Decision structure entropy: Hierarchy	R4
	Decision structure entropy: Network	R5
	Decision structure entropy: Hub	R6
Model 2: Pooling Neonatal HC/MC with OBS Nursery Wards	Queuing network analysis (i.e., under/overutilized capacity and number of days with overutilized capacity)	R7
	Arrival (max) entropy	R8
	Positional (max) entropy	R9
	Decision structure entropy: Hierarchy	R10
	Decision structure entropy: Network	R11
	Decision structure entropy: Hub	R12
Model 3: Pooling Neonatal Post-IC with Neonatal HC/MC	Queuing network analysis (i.e., under/overutilized capacity and number of days with overutilized capacity)	R13
	Arrival (max) entropy	R14
	Positional (max) entropy	R15
	Decision structure entropy: Hierarchy	R16
	Decision structure entropy: Network	R17
	Decision structure entropy: Hub	R18
Model 4 pooling Neonatal Post-IC with OBS Nursery Wards (Pooling Neonatal Post-IC with Neonatal HC/MC)	Queuing network analysis (i.e., under/overutilized capacity and number of days with overutilized capacity)	R19
	Arrival (max) entropy	R20
	Positional (max) entropy	R21
	Decision structure entropy: Hierarchy	R22
	Decision structure entropy: Network	R23
	Decision structure entropy: Hub	R24

**Table 2:** Utilization results. As for units NI, N2, and N3, the arrival rate (*λ*) is the effective arrival rate at the department (Eff λi) based on Formula (1). *c* is the average staffed beds that each unit has and *µ* is the service rate in each unit. The ρ (i.e., average utilization) is calculated using the traffic formula, as given in Formula (2).

**Delivery Room (O3)**		Neonatal IC Unit (N1)		Neonatal Post-IC Unit (N2)		Neonatal HC/MC Unit (N3)		Nursery Wards (O1)	
*c*	6	*c*	14	*c*	4	*c*	11	*c*	7
$\lambda_{O3}$	4.0	$\lambda_{N1}$	1.2	$\lambda_{N2}$	0.2	$\lambda_{N3}$	0.9	$\lambda_{O1}$	2.7
$\mu_{O3}$	1.00	$\mu_{N1}$	0.10	$\mu_{N2}$	0.06	$\mu_{N3}$	0.19	$\mu_{O1}$	0.91
ρ	67%	ρ	85%	ρ	96%	ρ	43%	ρ	43%

**Table 3:** Correlation analysis for the downstream wards. This is calculated using the Pearson correlation coefficient function in Excel.

	Post-IC Unit (N2)	HC/MC Unit (N3)	Wards (O1)
**Post-IC Unit (N2)**	1		
**HC/MC Unit (N3)**	0.09	1	
**Wards (O1)**	−0.03	0.22	1

**Table 4:** Output for Algorithm 1: the number of days with overutilized staffed beds.

Capacity Interventions	Variable	Average Overutilized Beds/Inpatient Wards (±SD)	Average Underutilized Beds/Inpatient Wards (±SD)	Number of Days with Overutilized Staffed Beds
Model 1 Capacity/department	R1	−0.4 (±0.7)	12 (±4)	166
Model 2 Pooling Neonatal HC/MC Unit (N3) with OBS Nursery Wards (O1)	R7	−0.7 (±1.2)	7.3 (±5)	182
Model 3 Pooling Neonatal Post-IC Unit (N2) with Neonatal HC/MC Unit (N3)	R13	−1.5 (±1.4)	11 (±5)	69
Model 4 Pooling Neonatal Post-IC Unit (N2) with OBS Nursery Wards (O1)	R19	−0.3 (±0.8)	14 (±3)	80

**Table 5:** Output of Algorithm 2: arrival entropy.

		Arrival Entropy	
Capacity Interventions	Variable	Arrival Entropy	Arrival Max Entropy
Model 1 Capacity/department (current situation)	R2	1.9	2.32
Model 2 Pooling Neonatal HC/MC Unit (N3) with OBS Nursery Wards (O1)	R8	1.24	2
Model 3 Pooling Neonatal Post-IC Unit (N2) with Neonatal HC/MC Unit (N3)	R14	1.82	2
Model 4 Pooling Neonatal Post-IC Unit (N2) with OBS Nursery Wards (O1)	R20	1.85	2

**Table 6:** Output of Algorithm 3: positional entropy.

		Positional Entropy	
Capacity Interventions	Variable	Positional Entropy	Positional Max Entropy
Model 1 Capacity/department (current situation)	R3	3.68	4.16
Model 2 Pooling Neonatal HC/MC Unit (N3) with OBS Nursery Wards (O1)	R9	3.09	3.58
Model 3 Pooling Neonatal Post-IC Unit (N2) with Neonatal HC/MC Unit (N3)	R15	2.2	2.58
Model 4 Pooling Neonatal Post-IC Unit (N2) with OBS Nursery Wards (O1)	R21	2.32	4.16

**Table 7:** The number of nurses scheduled per day for newborns. As for the Delivery Room (O3), the nurses are not exclusively caring for newborns but also the mothers.

Shifts	N1	N2	N3	O1	O3
Day	12	2	4	1	3
Night	8	2	2	1	3
Evening	7	2	2	1	3
Total	27	6	8	3	9

**Table 8:** Output of Algorithm 4: the decision-making structure entropy, given the local information.

	Decision-Making Structure Entropy		
Capacity Interventions	Hierarchy	Networks	Hub
Model 1 Capacity/department (current situation)	11.6	18	8
Model 2 Pooling Neonatal HC/MC Unit (N3) with OBS Nursery Wards (O1)	10.9	14.7	8.4
Model 3 Pooling Neonatal Post-IC Unit (N2) with Neonatal HC/MC Unit (N3)	10.6	13.7	7.4
Model 4 Pooling Neonatal Post-IC Unit (N2) with OBS Nursery Wards (O1)	11.2	15	8.6

**Table 9:** Output of Algorithm 4: the decision-making structure entropy, given aggregated information.

	Decision-Making Structure Entropy		
Capacity Interventions	Hierarchy + Aggregated Information	Networks + Aggregated Information	Hub + Aggregated Information
Model 1 Capacity/department (current situation)	3.6	10	0
Model 2 Pooling Neonatal HC/MC Unit (N3) with OBS Nursery Wards (O1)	2.6	6.4	0
Model 3 Pooling Neonatal Post-IC Unit (N2) with Neonatal HC/MC Unit (N3)	3	6.4	0
Model 4 Pooling Neonatal Post-IC Unit (N2) with OBS Nursery Wards (O1)	2.6	6.4	0

**Table 10:** Summary of entropy (bits) results, calculated with Formula (12).

Capacity Interventions	Without Aggregated Information			With Aggregated Information		
Capacity Interventions	Arrival (Max) + Positional (Max) Entropy + Hierarchy	Arrival (Max)+ Positional (Max) Entropy + Networks	Arrival (Max) + Positional (Max) Entropy + Hub	Arrival (Max) + Positional (Max) Entropy + Hierarchy	Arrival (Max)+ Positional (Max) Entropy + Networks	Arrival (Max) + Positional (Max) Entropy + Hub
Model 1 Capacity/department (current situation)	18.08	24.48	14.48	10.08	16.48	6.48
Model 2 Pooling Neonatal HC/MC Unit (N3) with OBS Nursery Wards (O1)	16.48	20.28	13.98	8.18	11.98	5.58
Model 3 Pooling Neonatal Post-IC Unit (N2) with Neonatal HC/MC Unit (N3)	15.18	18.28	11.98	7.58	10.98	4.58
Model 4 Pooling Neonatal Post-IC Unit (N2) with OBS Nursery Wards (O1)	17.36	21.16	14.76	8.76	12.56	6.16

## Data Availability

Not applicable.
